# *In silico* identification of the prognostic biomarkers and therapeutic targets associated with cancer stem cell characteristics of glioma

**DOI:** 10.1042/BSR20201037

**Published:** 2020-08-10

**Authors:** Wen Lvu, Xu Fei, Cheng Chen, Bo Zhang

**Affiliations:** Shenzhen People’s Hospital, Second Clinical Medical College of Jinan University, First Affiliated Hospital of Southern University of Science and Technology, 1017 Dongmen North Road, Luohu District, Shenzhen 518020, Guangdong, China

**Keywords:** Cancer stem cells, Glioma, mRNA expression-based stemness index, Prediction model, Prognostic biomarker

## Abstract

Glioma is the common histological subtype of malignancy in central nervous system, with a high morbidity and mortality. Cancer stem cells (CSCs) play an important role in regulating the tumorigenesis and progression of glioma; however, the prognostic biomarkers and therapeutic targets associated with CSC characteristics have not been fully acknowledged in glioma. In order to identify the prognostic stemness-related genes (SRGs) of glioma *in silico*, the RNA sequencing data of patients with glioma were retrieved from The Cancer Genome Atlas (TCGA) databases. The mRNA expression-based stemness index (mRNAsi) was significantly associated with the glioma histologic grade, isocitrate dehydrogenase 1 (IDH1) mutation and overall survival of glioma patients by the nonparametric test and Kaplan–Meier survival analysis. A total of 340 SRGs were identified as the overlapped stemness-related differential expressed genes (DEGs) of different histologic grade screened by the univariate Cox analysis. Based on 11 prognostic SRGs, the predict nomogram was constructed with the AUC of 0.832. Moreover, the risk score of the nomogram was an independent prognostic factor, indicating its significant applicability. Besides other eight reported biomarkers of glioma, we found that F2RL2, CLCNKA and LOXL4 were first identified as prognostic biomarkers for glioma. In conclusion, this bioinformatics study demonstrates the mRNAsi as a reliable index for the IDH1 mutation, histologic grade and OS of glioma patients and provides a well-applied model for predicting the OS for patients with glioma based on prognostic SRGs. Additionally, this *in silico* study also identifies three novel prognostic biomarkers (F2RL2, CLCNKA and LOXL4) for glioma patients.

## Introduction

Glioma is the most common histological subtype of primary tumor of central nervous system (CNS), accounting for approximately 80% of brain malignancies, with 15,000–17,000 new cases annually in the United States [1,2]. The lower-grade gliomas (LGGs) include the diffuse low-grade and intermediate-grade gliomas (World Health Organization [WHO] grades II and III), with a relatively favorable prognosis [3]. Glioblastoma (GBM) are generally more aggressive tumors (grade IV) with a higher morbidity and mortality [4]. Although advanced therapeutic strategy has been proposed, the 5-year overall survival (OS) rate of patients with GBM is still less than 5% [1]. Thus, identification the difference between LGGs and GBM, along with neoplasm histologic grade, may assist oncologists in finding the prognostic biomarkers and potential targets for the treatment of glioma.

The heterogeneity of tumor cells has long been appreciated and cancer stem cells (CSCs) are the important part of glioma [5]. Glioma CSCs are marked by common stem cell markers, such as CD133, CD15, CD44, Prominin-1, L1CAM and NPM1 [6]. They can work with immune niche factors and cellular microenvironment to regulate the tumorigenesis and progression of glioma [7]. During glioma treatment, CSCs are highly resistant to chemotherapy, radiation and immune recognition by regulating autophagy and proteases [8]. Thus, CSCs play important roles in glioma initiation, progression and therapeutic resistance. Identification of CSC-associated biomarkers may predict the tumor progression of glioma and provide CSC-based diagnostic and therapeutic strategies.

Nowadays, CSC characteristics have been identified to assess the oncogenic dedifferentiation by deep learning methods [9]. The DNA methylation-based stemness index (mDNAsi) can reflect the epigenetic stemness features, while the mRNA expression-based stemness index (mRNAsi) represents the transcriptomic stemness expression. It has been reported that mRNAsi is a reliable index in bladder cancer and is associated with tumorigenesis and tumor stage; however, its roles in glioma is still unclear.

In the present study, RNA-seq data and clinical information of patients with LGGs and GBM were collected from The Cancer Genome Atlas (TCGA) databases. Additionally, we identified the differential expressed genes (DEGs) and the association between mRNAsi and histologic grade, isocitrate dehydrogenase 1 (IDH1) mutation and OS. The stemness-related genes (SRGs) were also identified. Based on the prognostic SRGs, the predict model was constructed. Thus, this bioinformatics analysis provides potential prognostic biomarkers that may assist oncologists in clinical diagnosis and treatment of glioma.

## Method

### Data acquisition

Gene expression profiling of 705 primary brain gliomas including 532 LGGs and 173 GBMs were downloaded from The Cancer Genome Atlas (TCGA) (https://tcga-data.nci.nih.gov). The gene expression levels were downloaded in formats of Fragments Per Kilobase per Million (FPKM) and HTSeq-Counts. Clinical data of the demographics, tumor information and follow-up data of the all patients were also extracted from the database.

### Estimation of mRNAsi using OCLR

An algorithm named one-class logistic regression machine learning (OCLR) was reported by Tathiane et al. and could estimate the stemness signatures of the bulk tissue mRNA as an index called mRNAsi. Greater tumor dedifferentiation and higher activity of CSCs could be presented as higher mRNAsi [9]. In the present study, mRNAsi of LGG and GBM were estimated by this algorithm based on the normalized gene expression profile obtained from RNA-seq. The associations between mRNAsi and the OS, histologic grade and IDH1 were also established by nonparametric test and Kaplan–Meier survival analysis.

### DEGs analysis between low and high mRNAsi glioma

In the present study, EdgeR method was applied to identify the DEGs between experimental group and control group. All brain gliomas samples were divided into three groups by tumor grades (G1, G2 and G3). DEG analysis between high stemness tumors (samples with mRNAsi greater than the median) and low stemness tumors (samples with mRNAsi less than the median) were conducted in these three groups, respectively.

A gene with log2 Fold Change (FC) > 1.0 or < -1.0, and False Discovery Rate (FDR) value < 0.05 was defined as DEG. Furthermore, Kyoto Encyclopedia of Genes and Genomes (KEGG) and Gene Oncology (GO) functional enrichment analysis were used to explore the signaling pathways and biological processes that DEGs enriched [10].

### Identification of prognostic SRGs and construction of multivariate Cox regression model

The intersection of these three groups of DEGs were screened and included in the univariate Cox analysis. DEGs with significant statistical results were defined as prognostic SRGs and integrated into the initial multivariate Cox regression model. Then, the Least Absolute Shrinkage and Selection Operator (LASSO) regression was used to filter the independent variables essential to modeling and prevent model overfitting. In addition, the variables with non-zero coefficient in LASSO regression were included in the final multivariate model. Besides, the goodness of fit and the accuracy of the model was tested by residual plot and receiver operator characteristic (ROC) curve, respectively.

### Independent prognosis analysis

The risk score was calculated by the formula of the final multivariate Cox model (as followed) for each brain gliomas patients.

$${\text{RS}}_{x}\;=\beta 1\times {\text{gene}}_{1}+\beta 2\times {\text{gene}}_{2}\mathrm{+\beta}3\times {\text{gene}}_{3}\ldots \;\;\ldots +\beta \text{n}\times {\text{gene}}_{m}$$

In the formula, “β” represented coefficient of each prognostic gene; “*m*” represented the number of prognostic gene in the final multivariate model and “*x*” represented the number of each patient.

Moreover, all patients were divided into high and low risk group by the median of RS. The Kaplan–Meier survival curve was used to evaluate the prognosis value of RS. The univariate and multivariate Cox analysis, corrected by demographics and histologic grade, was used to evaluate the independent prognosis value of risk score.

### Construction and validation of the prognostic nomogram

The prognostic nomogram was constructed based on the multivariate Cox model including RS, which could predict the 3- and 5-year overall survival probability of brain gliomas patients. The calibration curve was used to evaluate the calibration of the nomogram. Besides, the RNA-seq and clinical data of Chinese Glioma Genome Atlas (CGGA) (http://www.cgga.org.cn/) were used to validate the generalizability of model in Asian populations [11].

### Statistics analysis

R software (Institute for Statistics and Mathematics, Vienna, Austria; www.r-project.org, version 3.6.1) were utilized for analysis. Two-sided *P* value < 0.05 was defined as statistically significant for all analysis process.

## Result

### DEGs analysis

**Figure 1:**
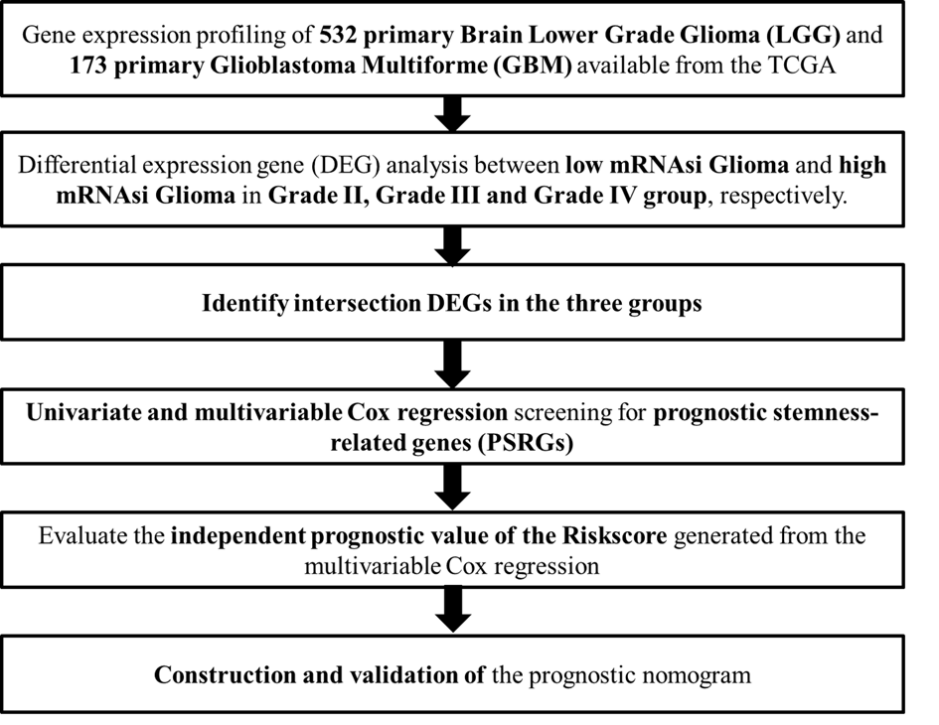
The flowchart of all analysis process

**Figure 2:**
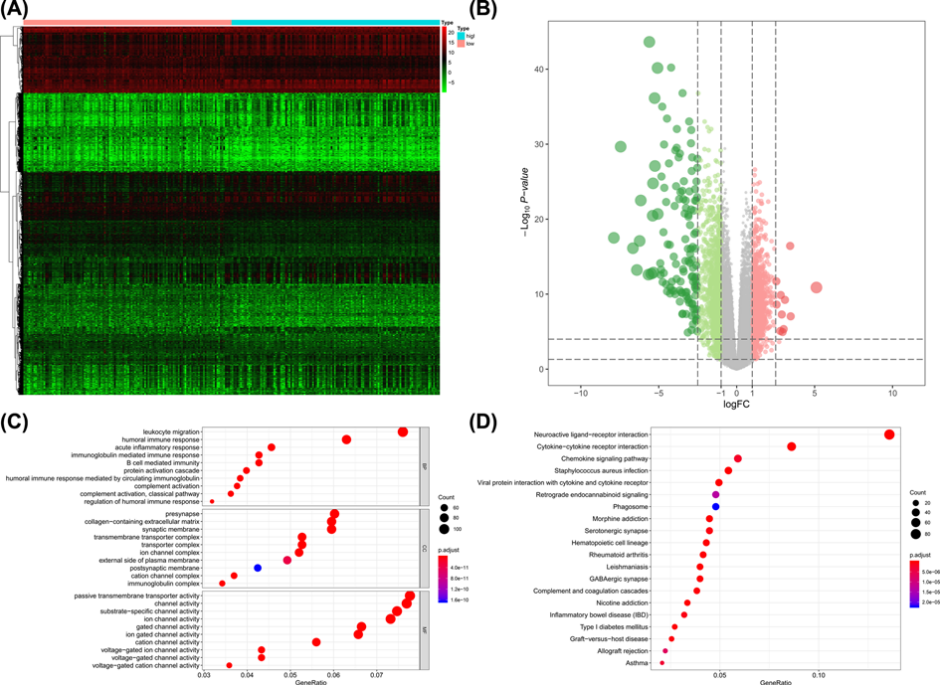
The results of differential expressed genes analysis and functional enrichment analysis between low mRNAsi tumors and high mRNAsi tumors in G2 brain gliomas The heatmap (**A**) and volcano plot (**B**) of the differential expressed genes. The GO (**C**) and KEGG (**D**) terms for the differential expressed genes.

The flowchart of the analysis process was summarized in Figure 1 and the baseline clinical characteristics of selected patients with glioma were summarized in Table 1. In G2 brain gliomas, a total of 2446 genes including 1335 down-regulated ones and 1111 up-regulated ones were identified as DEGs between low mRNAsi gliomas and high mRNAsi gliomas. The heatmap and volcano plot were presented in Figure 2A,B, respectively. The DEGs enriched in the GO term pointed to leukocyte migration, passive transmembrane transporter activity and channel activity (Figure 2C), while those enriched in KEGG term were associated with neuroactive ligand–receptor interaction (Figure 2D).

**Table 1:** Baseline information of 657 patients diagnosed with brain glioma

Variables	Total patients (*N*=657)
**Age, years**	
Mean ± SD	46.88 ± 15.40
Median (range)	46 (14–89)
Unknown	2 (0.30%)
**Gender**	
Female	276 (42.01%)
Male	379 (57.69%)
Unknown	2 (0.30%)
**Histologic grade**	
G2	242 (36.84%)
G3	262 (39.88%)
G4	151 (22.98%)
Unknown	2 (0.30%)
**TCGA project**	
Brain Lower Grade Glioma	506 (77.02%)
Glioblastoma Multiforme	151 (22.98%)

**Abbreviations:** SD, Standard deviation.

**Figure 3:**
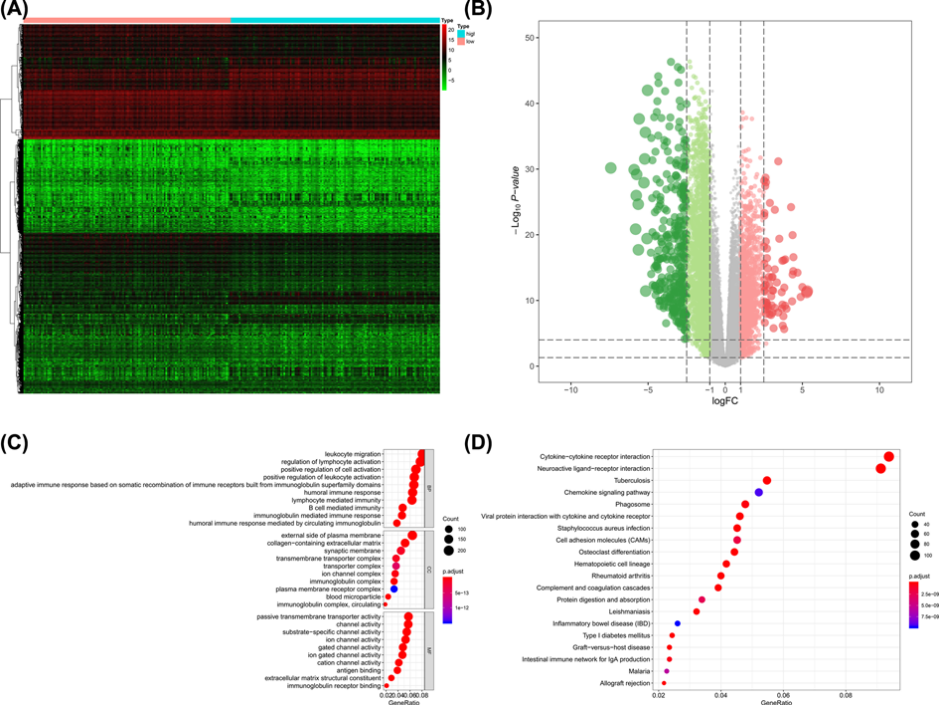
The results of differential expressed genes analysis and functional enrichment analysis between low mRNAsi tumors and high mRNAsi tumors in G3 brain gliomas The heatmap (**A**) and volcano plot (**B**) of the differential expressed genes. The GO (**C**) and KEGG (**D**) terms for the differential expressed genes.

Similarly, in G3 brain gliomas, 4629 genes including 3175 down-regulated ones and 1454 up-regulated ones were identified as DEGs between low mRNAsi gliomas and high mRNAsi gliomas. The heatmap and volcano plot were presented in Figure 3A,B, respectively. The significant GO (Figure 3C) and KEGG (Figure 3D) terms of stemness-related DEGs also included leukocyte migration, passive transmembrane transporter activity and neuroactive ligand–receptor interaction, along with external side of plasma membrane and cytokine–cytokine receptor interaction.

**Figure 4:**
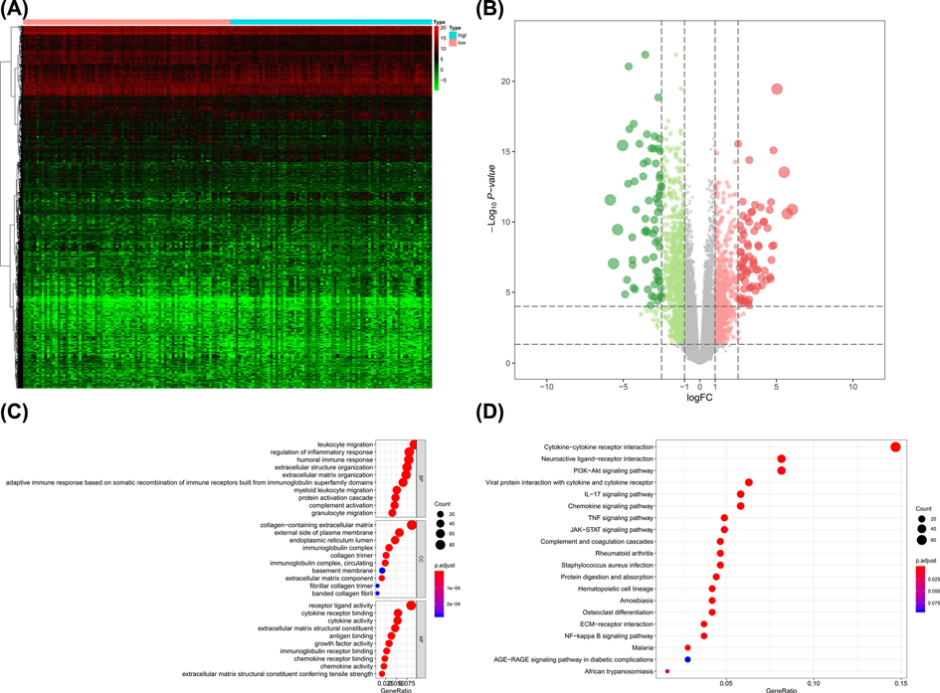
The results of differential expressed genes analysis and functional enrichment analysis between low mRNAsi tumors and high mRNAsi tumors in G4 brain gliomas The heatmap (**A**) and volcano plot (**B**) of the differential expressed genes. The GO (**C**) and KEGG (**D**) terms for the differential expressed genes.

In G4 brain gliomas, a total of 1765 DEGs including 1032 down-regulated ones and 733 up-regulated ones. The heatmap and volcano plot were presented in Figure 4A,B, respectively. GO terms including leukocyte migration, collagen-containing extracellular matrix and receptor ligand activity (Figure 4C) and KEGG terms including cytokine–cytokine receptor interaction and neuroactive ligand–receptor interaction (Figure 4D) were also identified as significantly enriched items among stemness-related DEGs

### Identification of prognostic SRGs

**Figure 5:**
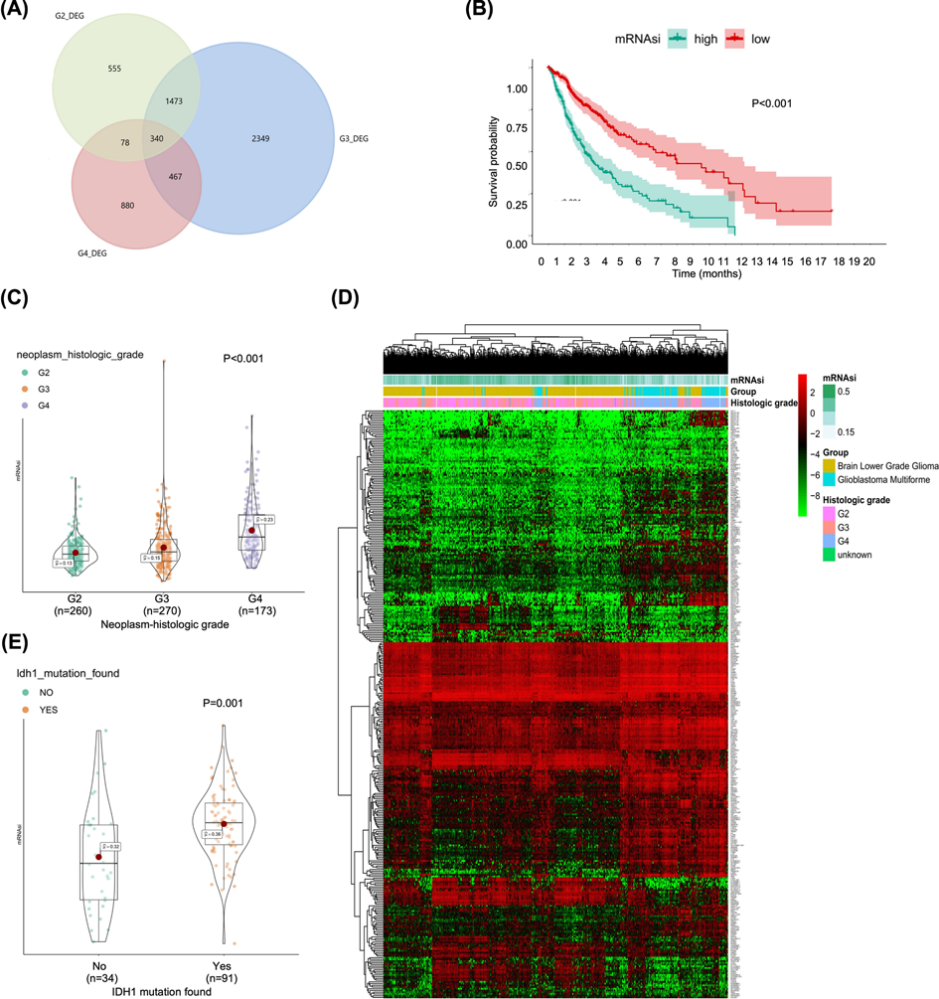
The clinical relevance of mRNAsi and identification of stemness-related genes (**A**) The Venn plot of the stemness-related differential expressed genes among histologic grade gliomas. The association between mRNAsi and prognosis (**B**), histologic grade gliomas (**C**) and IDH1 mutations (**D**). (**E**) The heatmap of the stemness-related differential expressed genes among different grade gliomas.

Among all the identified histologic grade DEGs, a total of 340 stemness-related DEGs were explored as the overlapped genes, which were regarded as SRGs (Figure 5A). In addition, mRNAsi of glioma were significantly associated with prognosis (Figure 5B, *P*<0.001). In order to identify the association between mRNAsi and important indexes, we used the nonparametric test and found that there was significant difference in mRNAsi among different histologic grade gliomas (Figure 5C, *P*<0.001). Furthermore, IDH1 mutations were more often detected in high mRNAsi tumors (Figure 5D, *P*=0.001). The 340 SRGs in different histologic grade gliomas were integrated into the univariate Cox analysis to identified key SRGs and we found that CCL7, CXCL6, SELE, C2CD4A, GPR141, TNFSF14, CCR2, GPR171, PTGER2 and VGLL3 were associated with histologic grade (Figure 5E).

### Identification of the prognostic model

**Figure 6:**
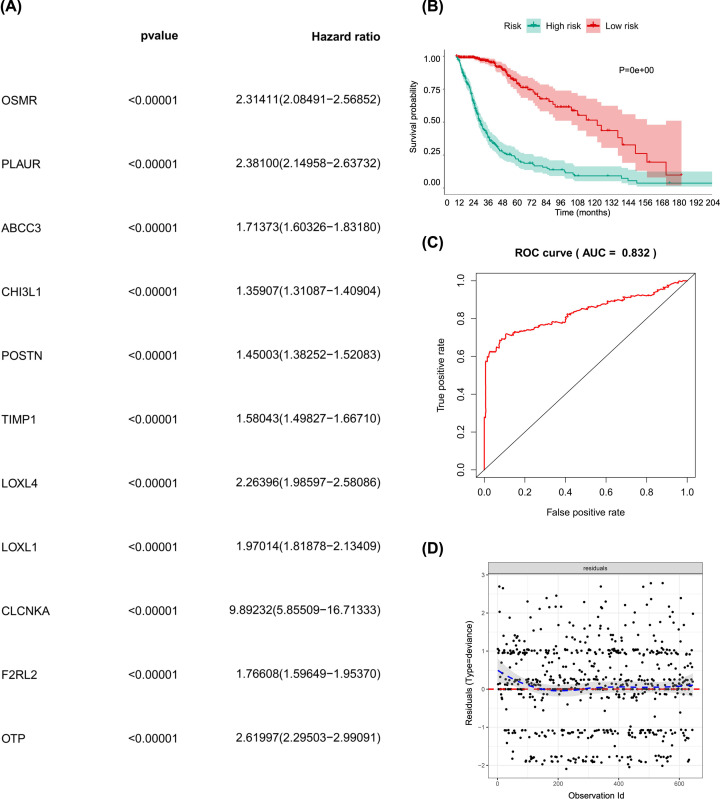
The model diagnosis of multivariate Cox model including prognostic stemness-related genes (**A**) The final multivariate model of the 11 prognostic stemness-related genes. (**B**) The Kaplan–Meier analysis of the risk score. The ROC curve (**C**) and residual plot (**D**) of the multivariate model.

A total of 11 prognostic SRGs with both statistical significances in univariate Cox analysis and non-zero coefficient in LASSO regression were included in the final multivariate model (Figure 6A and Supplementary Figure S1A,B), namely ABCC3, CHI3L1, CLCNKA, F2RL2, LOXL1, LOXL4, OSMR, OTP, PLAUR, POSTN and TIMP1. The risk scatter plot (Supplementary Figure S1C) and risk curve (Supplementary Figure S1D) of the final multivariate model demonstrated the distribution of risk score among all patients. The Kaplan–Meier analysis suggested that the risk score was significantly associated with the OS of patients with glioma (Figure 6B, *P*<0.001). The ROC curve and residual plot illustrated a high efficiency (AUC = 0.832, Figure 6C) and the residual distribution of the multivariate model (Figure 6D).

### Independent prognosis analysis, construction and validation of the prognostic nomogram

**Figure 7:**
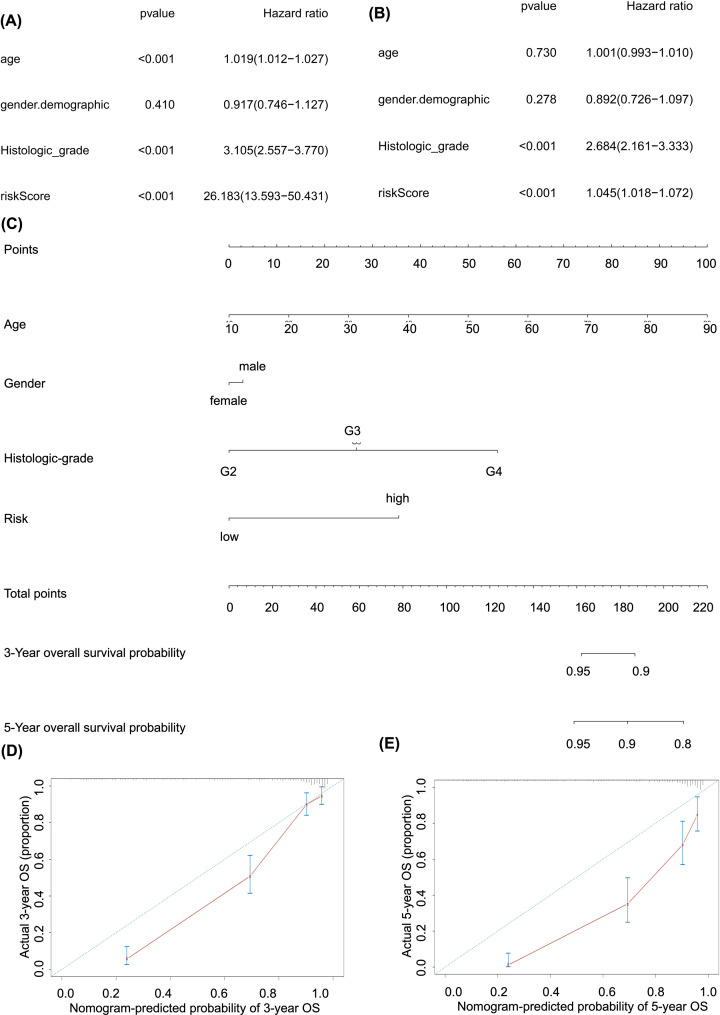
Independent prognosis analysis and construction of the prognostic nomogram The univariate (**A**) and multivariate (**B**) Cox regression model corrected by demographics and histologic grade. (**C**) The constructed prognostic nomogram based on the multivariate Cox model. (**D** and **E**) The calibration curve suggested acceptable calibration of the nomogram.

The risk score was shown to be an independently prognostic factor for glioma in both univariate (HR = 26.183, 95%CI (13.593−50.431), *P*<0.001) (Figure 7A) and multivariate (HR = 1.045, 95%CI (1.018−1.072), *P*<0.001) (Figure 7B) Cox regression model corrected by demographics and histologic grade. The prognostic nomogram was constructed based on the multivariate Cox model including risk score, which could predict the 3- and 5-year OS probability of brain gliomas patients (Figure 7C). The calibration curve suggested the acceptable calibration of the nomogram (Figure 7D,E).

### Identification of the novel prognostic biomarkers

Finally, the 11 prognostic SRGs in the final multivariate model were validated by the CGGA data and we found three novel prognostic biomarkers (F2RL2, CLCNKA and LOXL4) for glioma that have not been reported before (Supplementary Figures S2–4). All of them were associated with the OS of patients with primary glioma significantly (Supplementary Figure S2). In addition, all of them except LOXL4 were related to the OS of patients with recurrent glioma significantly (Supplementary Figures S2–4). As for these 11 prognostic SRGs, ABCC3 (Supplementary Figure S5) and POSTN (Supplementary Figure S6) were significantly associated with almost all subtypes of glioma. To minimize bias, external databases of The Human Protein Atlas [12] was also used to detect protein expression of 11 prognostic SRGs at the tissue level of brain and glioma (Supplementary Figure S7).

## Discussion

Advanced knowledge of glioma genome and proteome has promoted the identification of biomarkers to facilitate the prognosis prediction, early diagnosis and personalized treatment decisions [13–15]. However, due to the aggressive nature and therapeutic resistance, the treatment of glioma, in especial GBM, is still the dilemma for neurosurgeons and oncologists [8]. In the treatment resistance of radiotherapy, chemotherapy and even immunotherapy, the glioma CSCs play important roles. Thus, exploring the CSCs features of glioma may provide prognostic biomarkers and therapeutic targets for patients with glioma. Here, we found mRNAsi was a reliable index that was significantly up-regulated in high grade glioma and LGG with IDH1 mutation by bioinformatics methods. We developed a prediction model based on the prognostic SRGs that was well-applied in predicting the prognosis of glioma patients. Additionally, we also identified three novel prognostic biomarkers (F2RL2, CLCNKA and LOXL4) for glioma patients *in silico*.

Generally, the CSCs underlie glioma initial growth and recurrence following treatment-resistance [16]. Nowadays, the single-cell RNA-seq (scRNA-seq) has been applied in glioma to explore the distinct CSC populations [16]. It was found that most cancer cells are differentiated from the glial lineages (oligodendrocyte-like or astrocyte-like cells) and a subgroup of cells appear undifferentiated neural stem/progenitor cells which is responsible for fueling the glioma growth [17]. In another scRNA-seq study of IDH-mutant gliomas, CSCs driven the glioma differentiation and targeting them has a major impact on the tumor management [3].

Due to the significate roles of CSSs in the glioma, exploring the CSCs characteristics may provide potential biomarkers for progression monitor and therapeutic targets in the management of glioma. The CSCs characteristics are often governed by the epigenetic state, along with the activity of transcription factors (TFs), chromatin regulators and microenvironment cell networks [16]. In the present study, we used the mRNAsi to describe the CSCs characteristics of transcriptome gene expression. We found that the mRNAsi of glioma patients was significantly associated with pathological grade, IDH1 mutation and their OS.

The metabolic gene IDH1 is commonly mutated in gliomas [18]. Appropriately 80–90% of LGGs and 5% of GBMs were identified in the discovery of brain tumors [19,20]. The IDH1 enzyme, located in the cytosol/ peroxisome, can promote the oxidative decarboxylation of isocitrate to alpha-ketoglutarate (a-KG) [21]. Its mutation is supposed to be an early event in glioma formation and can promote the proliferation and colony formation of normal human astrocyte cell normal human astrocyte cells [22]. Additionally, the IDH-driven epigenetic changes remain the glioma cells in a less differentiated or stem-like state, rendering them vulnerable to suffering additional oncologic events, such as the mutation of tumor suppressor protein 53 (TP53) [23]. Thus, IDH1 enzyme may be related to stemness which is consistent with the results of the present study.

Exploring the predictors may also assist neurosurgeons and oncologists in clinical decision-marking of glioma, and thus many previous studies work on them by integrating clinical information and genomic biomarkers [24,25]. However, none of them cover the CSC-related signatures and prognostic SRGs, which play significant roles in the tumorigenesis, tumor development and drug resistance. According to the pathway analysis, we found the highly enrichment of leukocyte migration and neuroactive ligand–receptor interaction. The leukocyte migration often regulates immunosuppressive microenvironment and the lymphocyte specific protein 1 (LSP1)-induced immunosuppressive microenvironment has been reported to contribute to GBM [26]. In addition, CD133^+^ and Nestin^+^ glioma stem-like cells could also regulate SDF-1α and CXCR4, which subsequently promote leukocyte migration and glioma progression [27]. Generally, the tumorigenesis and progression of glioma is significantly with its microenvironment. The microenvironment cells interact with glioma cells with neuroactive ligand–receptor interaction [28,29].

In the present study, we identified eleven key prognostic SRGs, namely ABCC3, CHI3L1, CLCNKA, F2RL2, LOXL1, LOXL4, OSMR, OTP, PLAUR, POSTN and TIMP1. Based on the prognostic SRGs, we built a prediction model for glioma patients with a high accuracy and applicability (AUC: 0.832). Thus, the present study is a good supplement to the existing research about the prognosis evaluation of patients with glioma. In the prediction models, the identified key prognostic SRGs could also work as biomarkers for the OS and recurrence of patients with glioma. In the CGGA external validation, ABCC3 and POSTN were significantly associated with the OS in almost all the grades of primary and recurrent glioma. As one of the ATP-Binding Cassette (ABC) transporters, ABCC3 (also known as MRP3) is commonly overexpressed in many cancer types and mediates tumorigenesis, growth and chemoresistance by interacting with microenvironment cells [30–33]. In patients with glioma, some studies have also identified ABCC3 as a prognostic biomarker using microarray and next generation sequencing [34,35]. Along with ABCC3, POSTN (periostin) was also identified to be the important regulator in the tumorigenesis and treatment of glioma [36,37]. Other prognostic SRGs are also tightly involved in the development of many tumors, in especial glioma. For example, TIMP1 can regulate the alternative protease-independent activities [38,39]. The OSMR (receptor for the cytokine Oncostatin M) is a regulator of brain tumor stem cells (BTSC) proliferation by mediating EGFR phosphorylation [40]. CHI3L1 can be associated with IDH status and 1p/19q co-deletion in patients with glioma [41].

F2RL2, CLCNKA and LOXL4 are novel prognostic biomarkers for glioma which have not reported before. Their efficacies were also verified by external CGGA database. Compared with CLCNKA and LOXL4, F2RL2 was specifically applied in all grades of primary glioma, revealing its prognostic role in primary glioma. F2Rl2 is a G-protein-coupled receptor (GPCR) encoding the protease-activated receptor-3 (PAR3), which plays a clear role in inflammatory reactions and immune responses, and PAR3 can regulate the tumorigenesis and metastasis in many kinds of tumors including glioma [42–45]. Thus, we supposed that the roles of F2Rl2 in primary glioma might be related to PAR3 by inflammatory reactions. In addition, CLCNKA has been reported to mediate chloride channel and found to be dysregulated in the heart failure and salt-sensitive hypertension [46–48]. However, its roles in tumorigenesis are still unknown. LOXL4, encoding a member of the lysyl oxidase gene family, catalyzes oxidative deamination of lysine residues in collagen and elastin [49]. It takes part in cancer occurrence and metastasis in many tumors via p53 or FAK/Src pathway and its high expression is often associated with poor prognosis [49–52]. According to our results, we suppose that these three prognostic biomarkers potentially regulate the tumorigenesis and progression of brain glioma by mediating the CSCs.

## Conclusion

This bioinformatics analysis demonstrates the mRNAsi as a reliable index for the IDH1 mutation, histologic grade and OS of glioma and provides a well-applied model for predicting the OS for patients with glioma based on prognostic SRGs. Additionally, this *in silico* study also identifies three novel prognostic biomarkers (F2RL2, CLCNKA and LOXL4) for glioma patients.

## Supplementary Material

Supplementary Figures S1-S7

## Data Availability

The datasets analyzed in this study are available in The Cancer Genome Atlas (TCGA) and Chinese Glioma Genome Atlas (CGGA).

## Abbreviations

CSC: cancer stem cell
DEG: differential expressed gene
GPCR: G-protein-coupled receptor
IDH1: isocitrate dehydrogenase 1
mRNAsi: mRNA expression-based stemness index
PAR3: protease-activated receptor-3
SRG: stemness-related gene
TCGA: The Cancer Genome Atlas
