# Supplementary material for: In silico identification of the prognostic biomarkers and therapeutic targets associated with cancer stem cell characteristics of glioma
Source: Biosci Rep. 2020 Aug 10;40(8):BSR20201037. doi: 10.1042/BSR20201037 (PMC7418212; doi:10.1042/BSR20201037)
Supplement: Supplementary Figures S1-S7 [file BSR-2020-1037_supp.pdf]

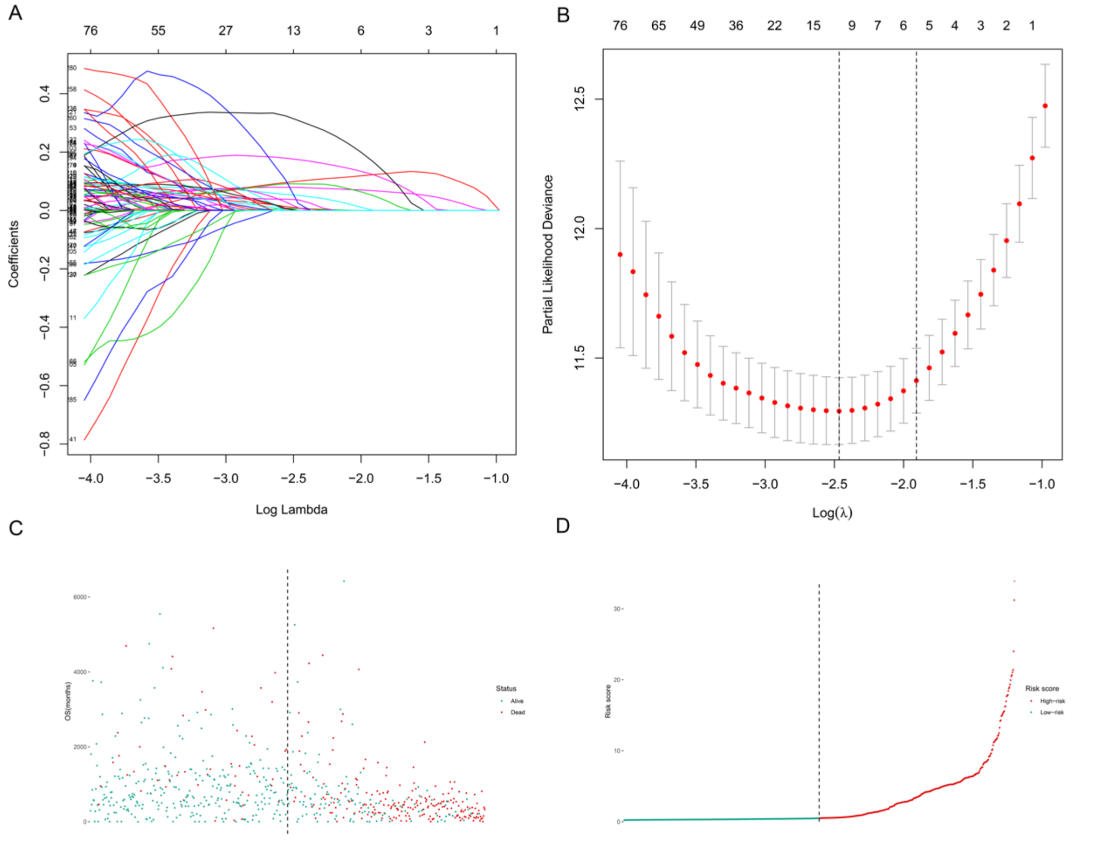

Figure S1 The construction of multivariate Cox model including prognostic stemness-related genes.

The univariate Cox analysis (A) and non-zero coefficient in LASSO regression (B) for the multivariate model including prognostic stemness-related genes. The risk scatter plot (C) and risk curve (D) of the multivariate model demonstrated the distribution of risk score among all the patients.

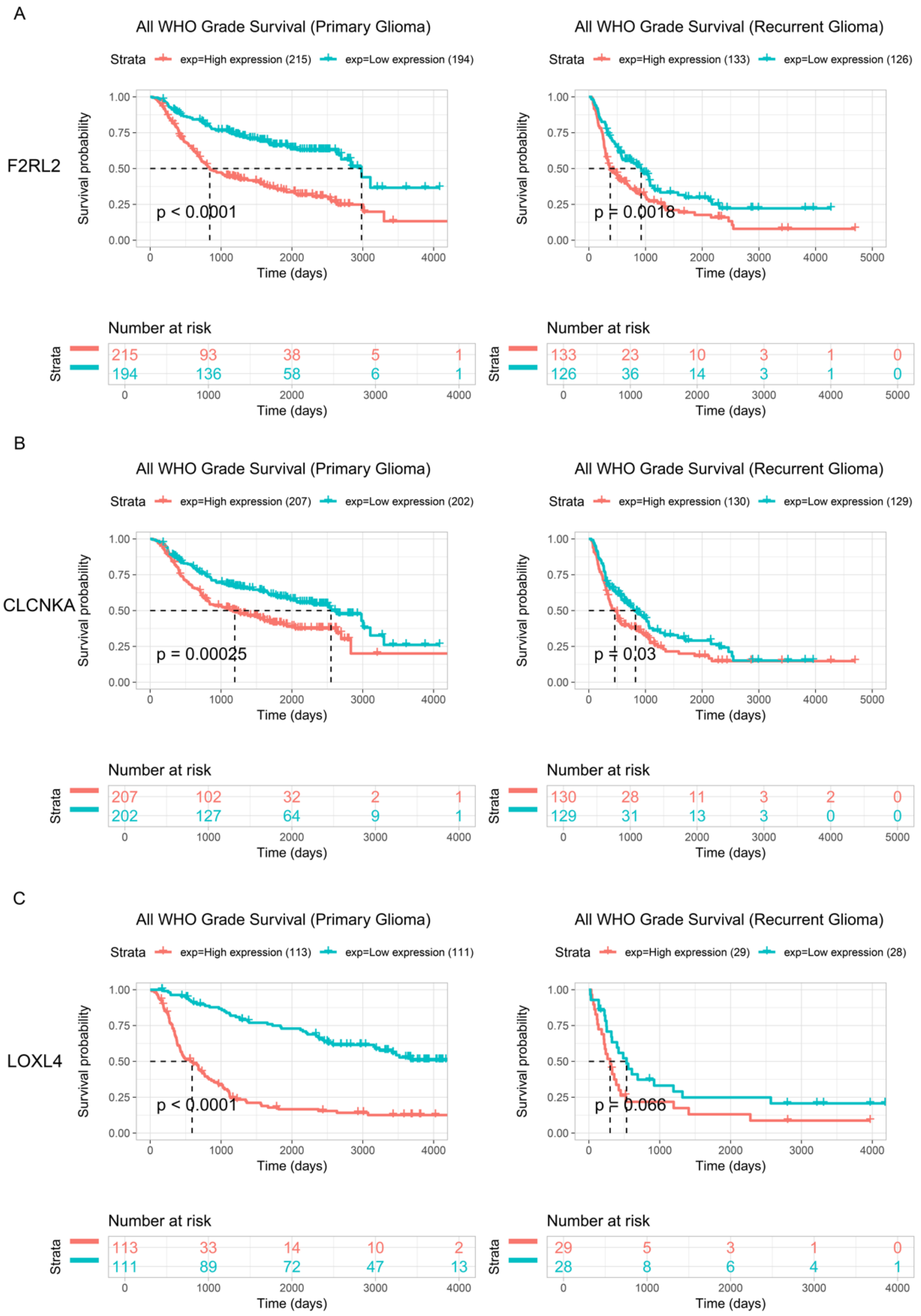

Figure S2 External validation of F2RL2, CLCNKA and LOXL4 by the Chinese Glioma Genome Atlas data.

The Kaplan-Meier analysis of F2RL2 in patients with primary (A) and recurrent (B) glioma; The Kaplan-Meier analysis of CLCNKA in patients with primary (C) and recurrent (D) glioma; The Kaplan-Meier analysis of LOXL4 in patients with primary (E) and recurrent (F) glioma.

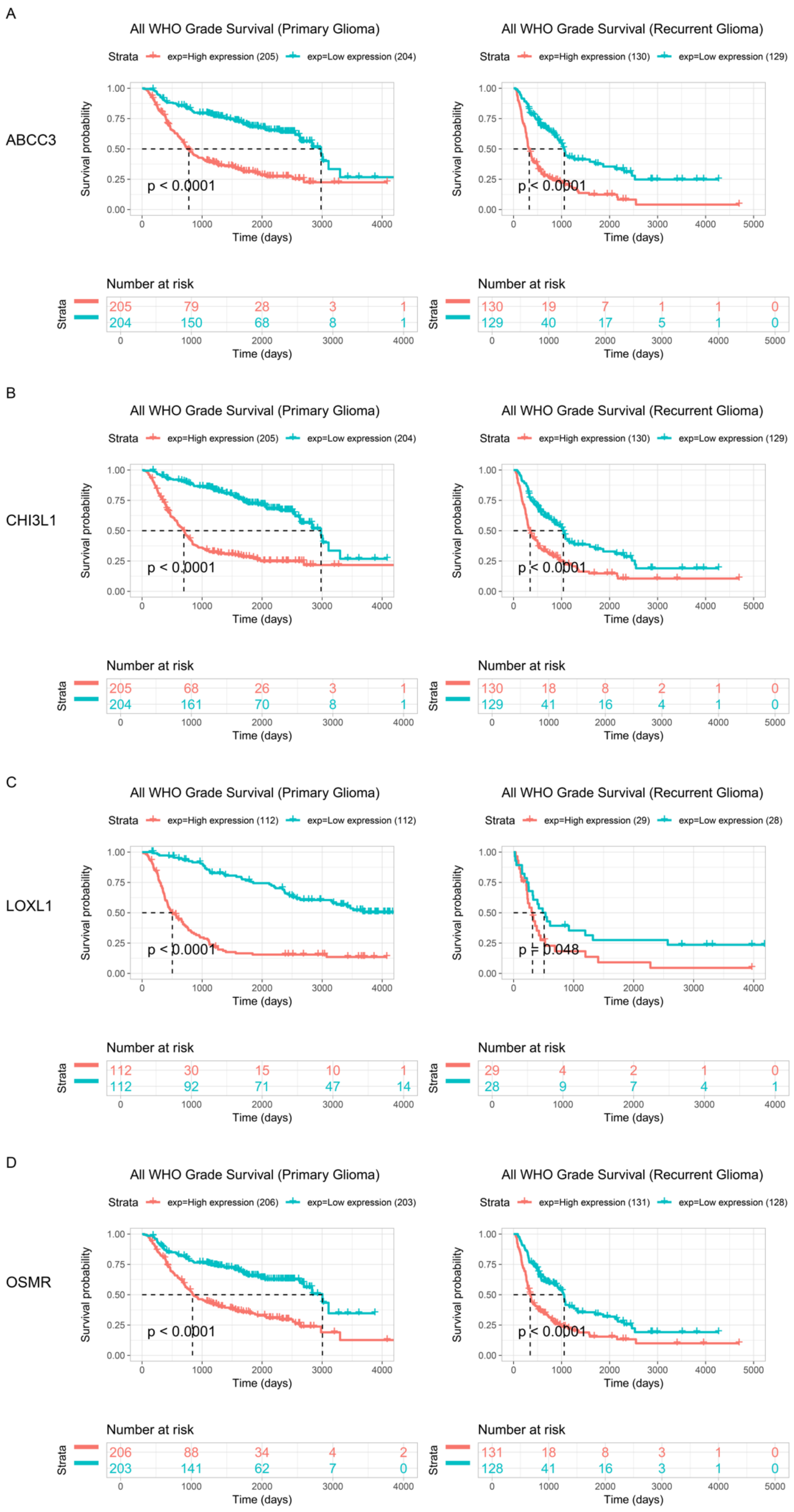
A
ABCC3
All WHO Grade Survival (Primary Glioma)
Strata exp=High expression (205) exp=Low expression (204)
p < 0.0001
Number at risk
205 79 28 3 1
204 150 68 8 1
All WHO Grade Survival (Recurrent Glioma)
Strata exp=High expression (130) exp=Low expression (129)
p < 0.0001
Number at risk
130 19 7 1 1 0
129 40 17 5 1 0
B
CHI3L1
All WHO Grade Survival (Primary Glioma)
Strata exp=High expression (205) exp=Low expression (204)
p < 0.0001
Number at risk
205 68 26 3 1
204 161 70 8 1
All WHO Grade Survival (Recurrent Glioma)
Strata exp=High expression (130) exp=Low expression (129)
p < 0.0001
Number at risk
130 18 8 2 1 0
129 41 16 4 1 0
C
LOXL1
All WHO Grade Survival (Primary Glioma)
Strata exp=High expression (112) exp=Low expression (112)
p < 0.0001
Number at risk
112 30 15 10 1
112 92 71 47 14
All WHO Grade Survival (Recurrent Glioma)
Strata exp=High expression (29) exp=Low expression (28)
p = 0.048
Number at risk
29 4 2 1 0
28 9 7 4 1
D
OSMR
All WHO Grade Survival (Primary Glioma)
Strata exp=High expression (206) exp=Low expression (203)
p < 0.0001
Number at risk
206 88 34 4 2
203 141 62 7 0
All WHO Grade Survival (Recurrent Glioma)
Strata exp=High expression (131) exp=Low expression (128)
p < 0.0001
Number at risk
131 18 8 3 1 0
128 41 16 3 1 0
Survival probability
Time (days)
Strata

Figure S3 External validation of ABCC3, CHI3L1, LOXL1, OSMR by the Chinese Glioma Genome Atlas data.

The Kaplan-Meier analysis of ABCC3 in patients with primary (A) and recurrent (B) glioma; The Kaplan-Meier analysis of CHI3L1 in patients with primary (C) and recurrent (D) glioma; The Kaplan-Meier analysis of LOXL1 in patients with primary (E) and recurrent (F) glioma; The Kaplan-Meier analysis of OSMR in patients with primary (G) and recurrent (H) glioma.

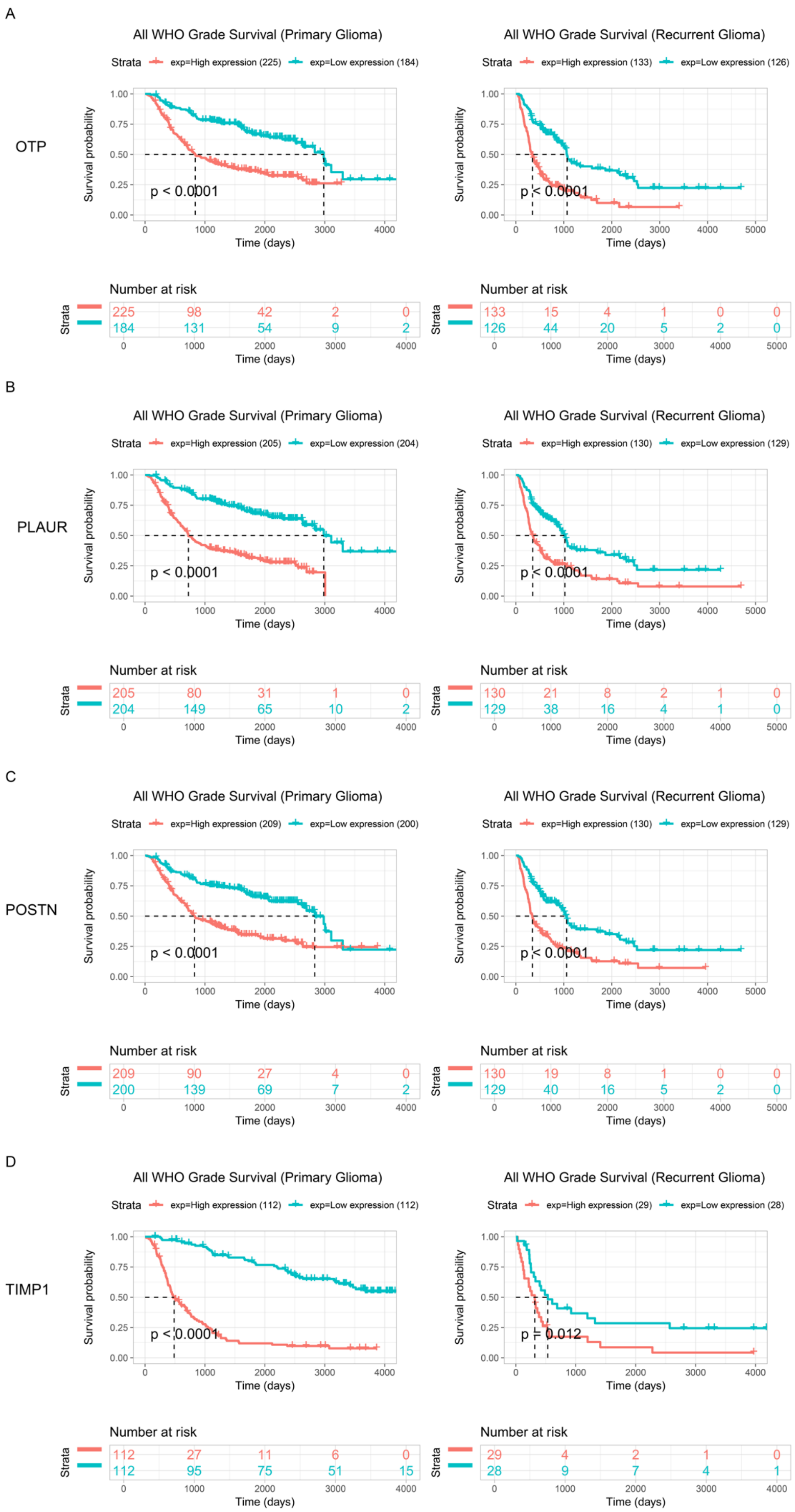
A
OTP
All WHO Grade Survival (Primary Glioma)
Strata exp=High expression (225) exp=Low expression (184)
p < 0.0001
Survival probability
Time (days)
Number at risk
225 98 42 2 0
184 131 54 9 2
All WHO Grade Survival (Recurrent Glioma)
Strata exp=High expression (133) exp=Low expression (126)
p < 0.0001
Number at risk
133 15 4 1 0 0
126 44 20 5 2 0
B
PLAUR
All WHO Grade Survival (Primary Glioma)
Strata exp=High expression (205) exp=Low expression (204)
p < 0.0001
Number at risk
205 80 31 1 0
204 149 65 10 2
All WHO Grade Survival (Recurrent Glioma)
Strata exp=High expression (130) exp=Low expression (129)
p < 0.0001
Number at risk
130 21 8 2 1 0
129 38 16 4 1 0
C
POSTN
All WHO Grade Survival (Primary Glioma)
Strata exp=High expression (209) exp=Low expression (200)
p < 0.0001
Number at risk
209 90 27 4 0
200 139 69 7 2
All WHO Grade Survival (Recurrent Glioma)
Strata exp=High expression (130) exp=Low expression (129)
p < 0.0001
Number at risk
130 19 8 1 0 0
129 40 16 5 2 0
D
TIMP1
All WHO Grade Survival (Primary Glioma)
Strata exp=High expression (112) exp=Low expression (112)
p < 0.0001
Number at risk
112 27 11 6 0
112 95 75 51 15
All WHO Grade Survival (Recurrent Glioma)
Strata exp=High expression (29) exp=Low expression (28)
p = 0.012
Number at risk
29 4 2 1 0
28 9 7 4 1
Strata

Figure S4 External validation of OTP, PLAUR, POSTN and TIMP1 by the Chinese Glioma Genome Atlas data.

The Kaplan-Meier analysis of OTP in patients with primary (A) and recurrent (B) glioma; The Kaplan-Meier analysis of PLAUR in patients with primary (C) and recurrent (D) glioma; The Kaplan-Meier analysis of POSTN in patients with primary (E) and recurrent (F) glioma; The Kaplan-Meier analysis of TIMP1 in patients with primary (G) and recurrent (H) glioma.

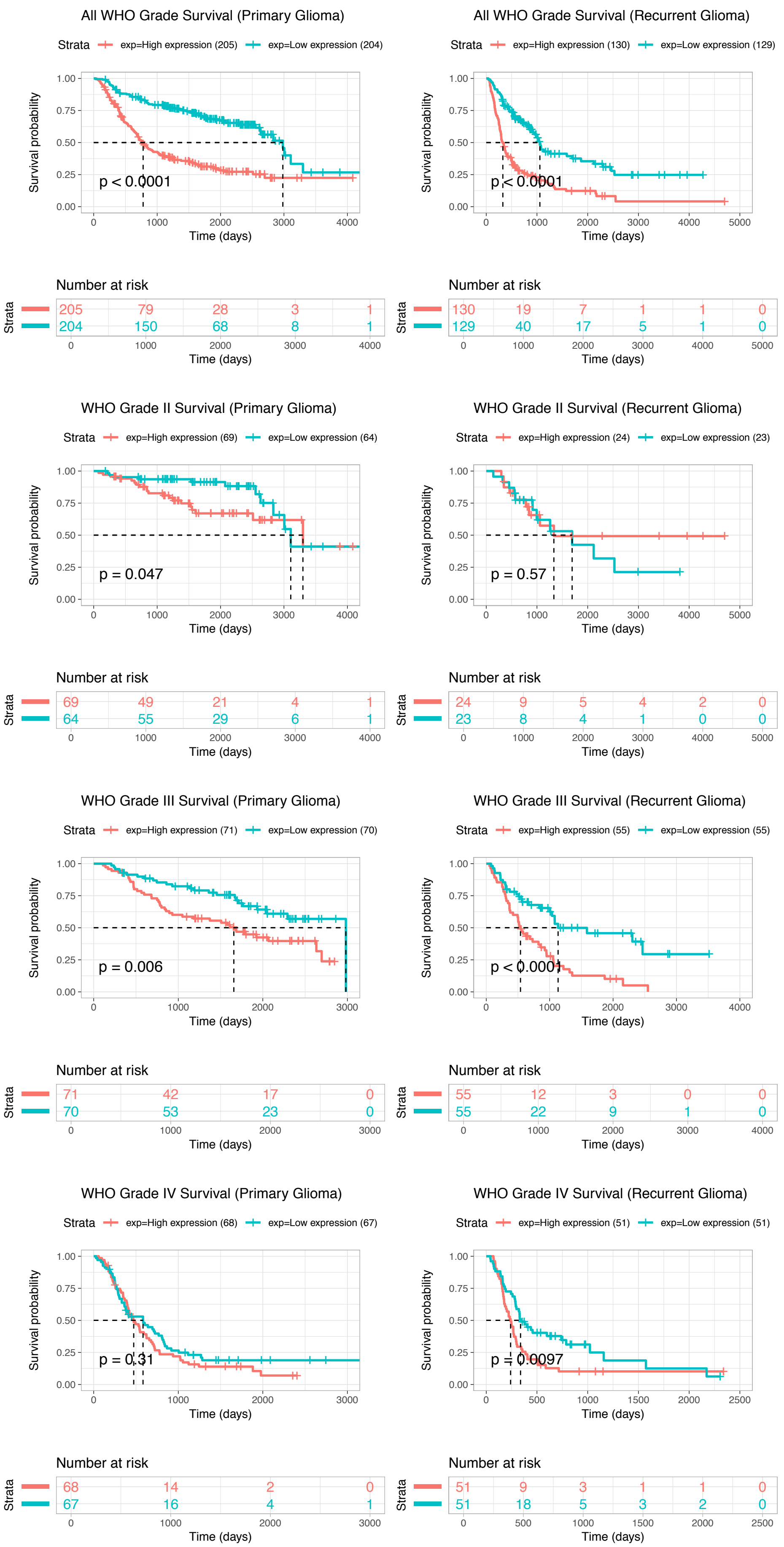
All WHO Grade Survival (Primary Glioma)
Strata exp=High expression (205) exp=Low expression (204)
Survival probability
p < 0.0001
Time (days)
Number at risk
205 79 28 3 1
204 150 68 8 1
All WHO Grade Survival (Recurrent Glioma)
Strata exp=High expression (130) exp=Low expression (129)
p < 0.0001
Number at risk
130 19 7 1 1 0
129 40 17 5 1 0
WHO Grade II Survival (Primary Glioma)
Strata exp=High expression (69) exp=Low expression (64)
p = 0.047
Number at risk
69 49 21 4 1
64 55 29 6 1
WHO Grade II Survival (Recurrent Glioma)
Strata exp=High expression (24) exp=Low expression (23)
p = 0.57
Number at risk
24 9 5 4 2 0
23 8 4 1 0 0
WHO Grade III Survival (Primary Glioma)
Strata exp=High expression (71) exp=Low expression (70)
p = 0.006
Number at risk
71 42 17 0
70 53 23 0
WHO Grade III Survival (Recurrent Glioma)
Strata exp=High expression (55) exp=Low expression (55)
p < 0.0001
Number at risk
55 12 3 0 0
55 22 9 1 0
WHO Grade IV Survival (Primary Glioma)
Strata exp=High expression (68) exp=Low expression (67)
p = 0.31
Number at risk
68 14 2 0
67 16 4 1
WHO Grade IV Survival (Recurrent Glioma)
Strata exp=High expression (51) exp=Low expression (51)
p = 0.0097
Number at risk
51 9 3 1 1 0
51 18 5 3 2 0

Figure S5 External validation of ABCC3 by the Chinese Glioma Genome Atlas data in different subtypes.

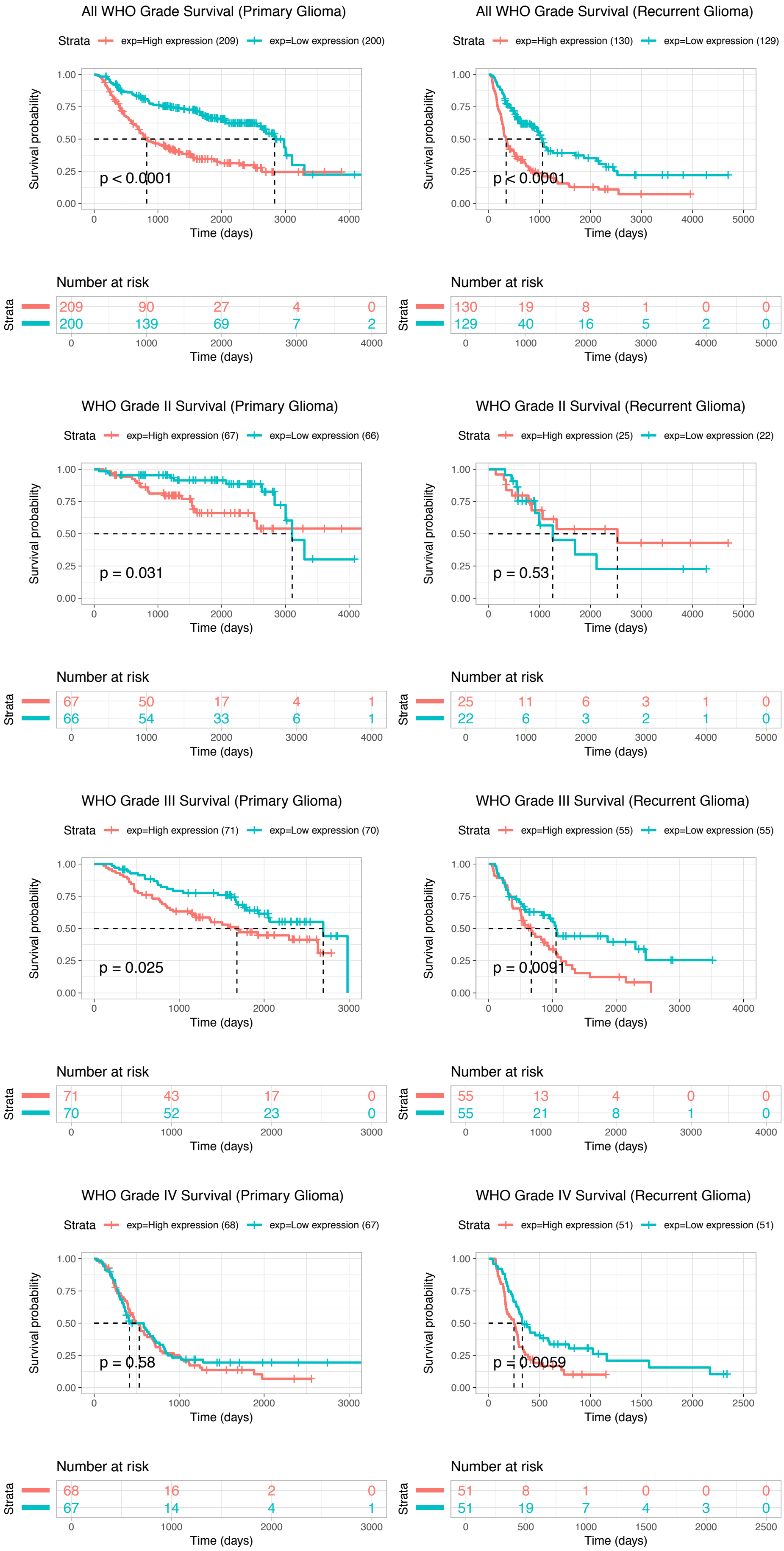
All WHO Grade Survival (Primary Glioma)
Strata exp=High expression (209) exp=Low expression (200)
Survival probability
Time (days)
p < 0.0001
Number at risk
209 90 27 4 0
200 139 69 7 2
All WHO Grade Survival (Recurrent Glioma)
Strata exp=High expression (130) exp=Low expression (129)
p < 0.0001
Number at risk
130 19 8 1 0 0
129 40 16 5 2 0
WHO Grade II Survival (Primary Glioma)
Strata exp=High expression (67) exp=Low expression (66)
p = 0.031
Number at risk
67 50 17 4 1
66 54 33 6 1
WHO Grade II Survival (Recurrent Glioma)
Strata exp=High expression (25) exp=Low expression (22)
p = 0.53
Number at risk
25 11 6 3 1 0
22 6 3 2 1 0
WHO Grade III Survival (Primary Glioma)
Strata exp=High expression (71) exp=Low expression (70)
p = 0.025
Number at risk
71 43 17 0
70 52 23 0
WHO Grade III Survival (Recurrent Glioma)
Strata exp=High expression (55) exp=Low expression (55)
p = 0.0091
Number at risk
55 13 4 0 0
55 21 8 1 0
WHO Grade IV Survival (Primary Glioma)
Strata exp=High expression (68) exp=Low expression (67)
p = 0.58
Number at risk
68 16 2 0
67 14 4 1
WHO Grade IV Survival (Recurrent Glioma)
Strata exp=High expression (51) exp=Low expression (51)
p = 0.0059
Number at risk
51 8 1 0 0 0
51 19 7 4 3 0

Figure S6 External validation of POSTN by the Chinese Glioma Genome Atlas data in different subtypes.

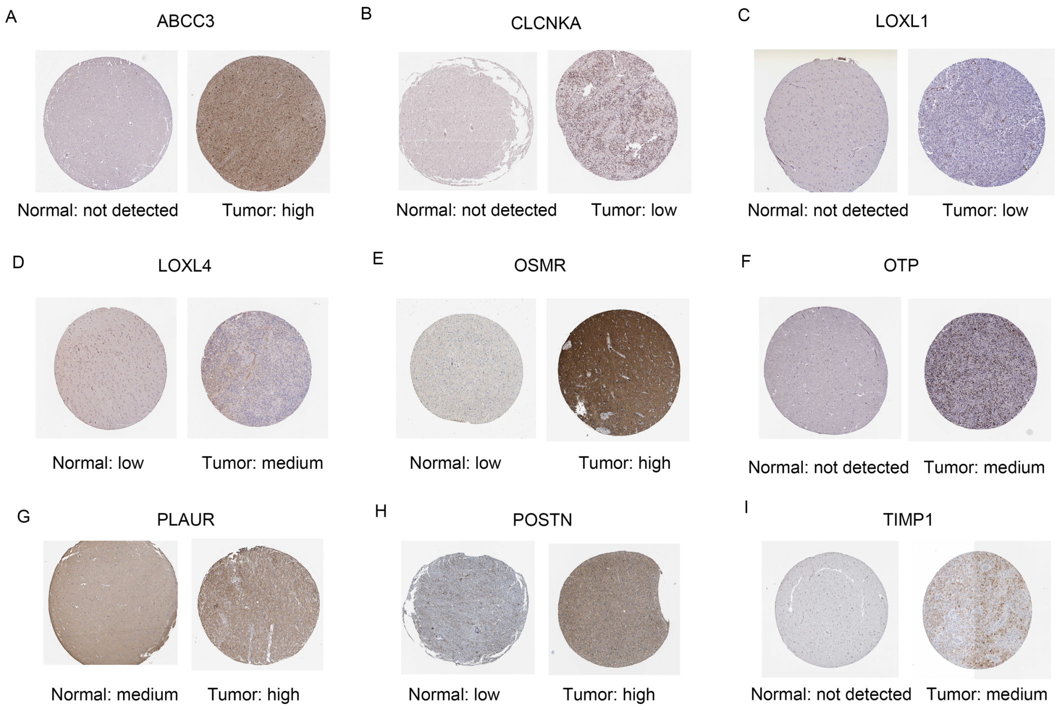

Figure S7 External validation of prognostic SRGs by the immunohistochemistry in the The Human Protein Atlas.
